# Supplementary figures and images for: Early versus deferred anti-SARS-CoV-2 convalescent plasma in patients admitted for COVID-19: A randomized phase II clinical trial
Source: PLoS Med. 2021 Mar 3;18(3):e1003415. doi: 10.1371/journal.pmed.1003415 (PMC7929568; doi:10.1371/journal.pmed.1003415)

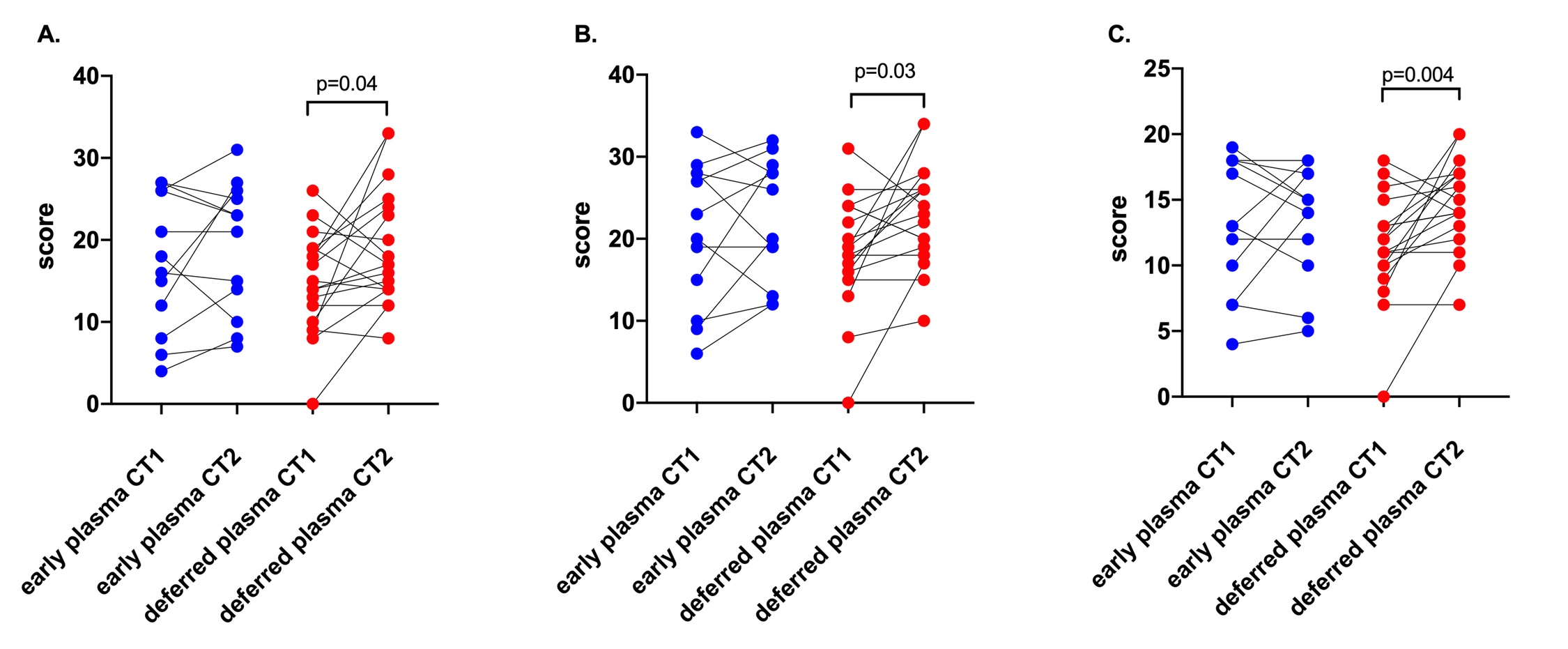

Supplement: S1 Fig — (A) CT score 1 (Zhou et al. [22]). (B) CT score 2 (Yang et al. [25]). (C) CT score 3 (Pan et al. [23,24]). (TIF) [file pmed.1003415.s001.tif]
