# Supplementary material for: Early versus deferred anti-SARS-CoV-2 convalescent plasma in patients admitted for COVID-19: A randomized phase II clinical trial
Source: PLoS Med. 2021 Mar 3;18(3):e1003415. doi: 10.1371/journal.pmed.1003415 (PMC7929568; doi:10.1371/journal.pmed.1003415)
Supplement: S2 Table — (DOCX) [file pmed.1003415.s003.docx]

**S2 Table.** Laboratory outcomes

|  | Early plasma group  (n=-28) | Deferred plasma group  (n=29) | P-value |
| --- | --- | --- | --- |
| **CRP**^a^ (mg/dl), median (IQR) (Nº) | | | |
| - day 0 | 9.2 (5.1-15.2) (28) | 9.5 (4.1-16.1) (27) | 0.81 |
| - day 3 | 5,3 (2.1-12.8) (24) | 7.7 (3.15-12.5) (25) | 0.39 |
| - day 7 | 3.9 (1.2-5.6) (16) | 3.2 (1.2-9.2) (20) | 0.94 |
| **Lymphocyte count** (x10^9^/L), median (IQR) (Nº) | | | |
| - day 0 | 680 (490-910) (25) | 800 (530-1120) (27) | 0.46 |
| - day 3 | 760 (540-1190) (23) | 620 (520-840) (24) | 0.15 |
| - day 7 | 760 (450-1040) (17) | 910 (500-1260) (19) | 0.66 |
| **IL-6** (pg/ml), median (IQR) (Nº) | | | |
| - day 0 | 46.3 (27.9-120.6) (28) | 53.3 (18.5-127.8) (25) | 0.88 |
| - day 3 | 23.6 (10.2-48.5) (24) | 20.8 (8-81.4) (26) | 0.86 |
| - day 7 | 42.9 (4.2-110.7) (17) | 14.8 (6-93.8) (19) | 1.00 |
| **Procalcitonin** (ng/ml), median (IQR) (Nº) | | | |
| - day 0 | 0.18 (0.09-0.73) (28) | 0.09 (0.08-0.20) (27) | 0.18 |
| - day 3 | 0.12 (0.09-0.31) (24) | 0.11 (0.08-0.24) (26) | 0.82 |
| - day 7 | 0.09 (0.08-0.21) (15) | 0.10 (0.08-0.14) (19) | 0.96 |
| **Ferritin** (ng/ml), median (IQR) (Nº) | | | |
| - day 0 | 881 (571-1714) (28) | 849 (599-1228) (28) | 0.58 |
| - day 3 | 1215 (589-1934) (24) | 1001 (572-2057) (26) | 0.78 |
| - day 7 | 1021 (520-1679) (16) | 974 (587-1556) (20) | 0.92 |
| **D-dimer** (ng/ml), median (IQR) (Nº) | | | |
| - day 0 | 1128 (658-1689) (28) | 926 (622-1133) (29) | 0.30 |
| - day 3 | 974 (614-1255) (24) | 835 (615-1326) (26) | 0.87 |
| - day 7 | 963 (723-2510) (15) | 948 (664-1987) (20) | 0.68 |
| **LDH** (U/L), median (IQR) (Nº) | | | |
| - day 0 | 317 (256-396) (28) | 299 (251-350) (27) | 0.35 |
| - day 3 | 331 (260-424) (24) | 320 (260-404) (25) | 0.78 |
| - day 7 | 290 (254-357) (17) | 317 (233-442) (20) | 0.58 |
| **HS Troponin T** (pg/ml), median (IQR) (Nº) | | | |
| - day 0 | 8.80 (6.30-7.25) (28) | 9.45 (7.25-19.3) (28) | 0.53 |
| - day 3 | 8.20 (5.33-14.1) (24) | 10.0 (6.35-16.4) (25) | 0.46 |
| - day 7 | 7.30 (5.53-17.9) (16) | 13.2 (6.10-24.4) (19) | 0.21 |
| **Pro-BNP** (pg/ml), median (IQR) (Nº) | | | |
| - day 0 | 150 (46.5-433) (28) | 166 (57.5-761) | 0.79 |
| - day 3 | 247 (117-775) (25) | 370 (132-1450) (25) | 0.55 |
| - day 7 | 149 (62-341) (16) | 336 (89-2492) (19) | 0.19 |
| CRP: C-reactive protein; IL-6: interleukin 6; LDH: lactate dehydrogenase; HS troponin T: high-sensitive troponin T; Pro-BNP: N-terminal (NT)-pro hormone B -type natriuretic peptide | | | |
