# Supplementary material for: Early versus deferred anti-SARS-CoV-2 convalescent plasma in patients admitted for COVID-19: A randomized phase II clinical trial
Source: PLoS Med. 2021 Mar 3;18(3):e1003415. doi: 10.1371/journal.pmed.1003415 (PMC7929568; doi:10.1371/journal.pmed.1003415)
Supplement: S3 Table — (DOCX) [file pmed.1003415.s004.docx]

**S3 Table.** Radiological changes (from day 0 to day 5) for early and deferred plasma groups, based on expert radiologist criteria

| Blinded expert criteria  (combined CT and chest X-ray analysis) | Early plasma group  (n=21) | Deferred plasma group  (n=24) | Effect estimate  (95% CI) | P value |
| --- | --- | --- | --- | --- |
| - Stable or improved  Nº(%) | 10 (47.6) | 13 (54.2) | OR 1.30  (95%CI 0.41- 3.89) | 0.77 |
| - Progressed (worse)  Nº(%) | 11 (52.4) | 11 (45.8) |  |  |
