# Supplementary material for: Early versus deferred anti-SARS-CoV-2 convalescent plasma in patients admitted for COVID-19: A randomized phase II clinical trial
Source: PLoS Med. 2021 Mar 3;18(3):e1003415. doi: 10.1371/journal.pmed.1003415 (PMC7929568; doi:10.1371/journal.pmed.1003415)
Supplement: S1 Text — (DOCX) [file pmed.1003415.s005.docx]

**S1 Text**

**STUDY PROTOCOL** and statistical analysis plan

**Early Anti-SARS-COV-2 Convalescent Plasma in Patients Admitted for COVID-19 Infection: A Randomized Phase II Trial**

Trial registration ClinicalTrials.gov Identifier: NCT04375098

After the study had begun, we adapted the protocol to add troponin and pro-BNP plasma level determination into the patient follow-up.

**Objectives**

General objective:

To evaluate the efficacy and safety of early convalescent plasma therapy on the progression of COVID-19.

Specific objectives:

- To study the efficacy of convalescent plasma in preventing COVID-19 clinical progression, comparing immediate plasma administration versus administration only at respiratory failure development or prolonged hospitalization.
- To study the safety of convalescent plasma in hospitalized patients with COVID-19.
- To determine the effect of convalescent plasma in the SARS-CoV-2 clearance rate in hospitalized patients with COVID-19.

**Study Design**

Prospective, single center, parallel-group, open-label randomized trial.

**Setting**

Medical unit of a Chilean academic tertiary center.

**Population**

**Inclusion criteria**

(1) Patients over 18 years old

(2) Hospitalized with COVID-19 symptoms present at enrolment and confirmed with a positive SARS-CoV-2 real-time polymerase chain reaction (RT-PCR) in nasopharyngeal swab

(3) COVID-19 symptoms ≤ 7 days to enrolment

(4) A CALL prediction score ≥ 9 points at enrolment* ^1^

(5) Eastern Cooperative Oncology Group (ECOG) performance status before SARS-CoV-2 infection 0-2.

**Exclusion criteria**

(1) PaO2/Fi O2 less than 200 or need for mechanical ventilation at enrolment

(2) Coinfection with other potentially relevant respiratory pathogen on admission

(3) Pregnancy or lactation

(4) Known IgA Nephropathy or IgA deficiency

(5) Previous intravenous immunoglobulin or plasma administration within the last 60 days

(6) Previous transfusion reactions that justify plasma contraindication (e.g. severe allergy)

(7) Do not resuscitate indication

(8) Patients participating in another interventional study related to COVID-19

(9) Patients that under investigator criteria had any condition that makes them unsuitable for study participation.

* CALL score includes the following parameters: age (< or > 60 years old), lymphocyte count (< or >1000), LDH (≤ 250; 250-500; >500) and comorbidities. The following comorbidities are included in the original CALL score: hypertension, diabetes, cardiovascular disease, liver disease, asthma, chronic lung disease, HIV infection and malignancy for at least 6 months. We added to comorbidities obesity (BMI≥ 30), chronic renal failure (stage III-V), solid organ transplant (<6 months) or hematopoietic transplant (<1 year), and immunosuppressant or immunomodulators use. We specified HIV infection with CD4<200 and cancer under current systemic treatment or in advance stage (IV).

CALL score is intended to predict the risk of developing severe COVID-19 defined as: SaO2≤ 93%, tachypnea ≥30 rpm, PaO2/FiO2 ≤ 300 o mechanical ventilation. Probability of progression to respiratory insufficiency is <10% for a CALL score 4-6, 10-40% for a CALL score 7-9 and >50% for a CALL score 10-13 (PPV 15.1; NPV 0.57) [20].

**Randomization**

Eligible patients will be randomly assigned via computer generated numbering by a block randomization sequence into two groups: early or deferred plasma transfusion. Randomization will be done by an independent member and sequence will be concealed to study investigators.

**Convalescent plasma donation protocol**

Plasma will be obtained from volunteer patients who have recovered from COVID-19, having been asymptomatic for at least 28 days, with a negative SARS-CoV-2 RT-PCR in nasopharyngeal swab and in plasma, and anti-SARS-CoV-2 (S1) IgG titers ≥ 1:400 (Elisa Euroinmun). Given that for the Euroimmun SARS-CoV-2 IgG ELISA, a positive assay (defined as a ratio of sample optical density [OD]/calibrator OD ≥ 1.1) is determined—as per the provider—with a basal dilution of 1:100, we decided to further semi-quantify the IgG in donor plasma with an additional fourth fold dilution, and established the 1:400 cutoff as the requirement for our plasma donors (considering again an OD ratio ≥ 1.1 as a positive result for that new dilution).

A sample of plasma will be cryopreserved in order to measure neutralizing antibodies titers later, as described below. Only male or nulliparous female donors will be included to avoid the risk of anti-HLA/HNA/HPA antibodies. Donor plasma will be tested for standard infectious diseases before administration (hepatitis B virus, hepatitis C virus, HIV, HTLV I/II, *Trypanosoma cruzi* and *Treponema pallidum* serologies, as well as NAAT for HIV, hepatitis B, and hepatitis C viruses). Extracted plasma will be immediately frozen at -20°C according to standard national safety measures.^2^

**SARS-CoV-2 RT- PCR:**

Nucleic acids for SARS-CoV-2 detection from plasma and nasopharyngeal samples will be automatically extracted using MagNA Pure System (Roche) accordingly to manufacturer’s instructions. Reverse-transcription polymerase chain reaction (RT-PCR) will be performed targeting SARS-CoV-2 RdRP gen, using LightMix 2019-nCoV Real-time RT-PCR kit (TibMolBiol) and LightCycler 480 (Roche) for amplification. A threshold cycle (CT) above 35 will be interpreted as an indeterminate^3^.

**Anti-SARS-CoV-2 IgG ELISA**

For specific IgG enzyme-linked immunosorbent assays (ELISA) we will use the commercial kit CE-marked Euroimmun (Lübeck, Germany, # EI 2606-9601 G), which uses the S1-domain of the spike protein of SARS-CoV-2 as antigen. Fresh or thawed serum samples will be first diluted at 1:101 as is recommended by the instructions, then 2-fold serial dilutions will be done until 1:6400. The following step will be followed according to the manufacturer's protocol. Samples' immunoreactivity will be measured at an optical density (OD) of 450 nm (OD450). Results will be expressed as a ratio between OD of patients’ sample and OD of the calibrator provided with each ELISA kit. The quantitative results obtained will be interpreted as follows: OD ratio: <0.8 = seronegative; ≥0.8 and < 1.1 = indeterminate; ≥1.1 = seropositive. The end-point dilution will be determined for each sample as the final dilution where the OD ratio (patient/calibrator) is ≥1.1. Seroconversion will be defined as seronegative at baseline and seropositive after 3 or 7 days or, a 4-fold increase in end-point dilution titer from the baseline.

**Neutralizing antibody titers assay**

Anti-SARS-CoV-2 neutralizing antibodies will be measured in plasma samples using an HIV-1 backbone expressing firefly luciferase as a reporter gene and pseudotyped with the SARS-CoV-2 Spike glycoprotein^4,5^. Samples with a neutralizing activity of at least 50% at a 1:160 dilution will be considered positive and used to perform titration curves and ID50 calculations. Determination of the ID50 will be performed using a 4-parameter nonlinear regression curve fit measured as the percent of neutralization determined by the difference in average relative light units (RLU) between test wells and pseudotyped virus controls. In order to perform the ID50 calculations, the lack of fit test will have a p-value > 0.1. The top values will be constrained to 100 and the bottom values will be set to 0.

**Intervention and comparison**

The early plasma group will receive convalescent plasma on the day of enrolment. The deferred plasma group will receive convalescent plasma only if a prespecified worsening respiratory function criterion is met during hospitalization (Pa02/Fi02 <200) or, if the patient still requires hospitalization for symptomatic COVID-19 >7 days after enrolment.

The transfusion will consider a total of 400 mL of ABO compatible convalescent plasma, constituted by two 200 mL units separated by 24 hours. Plasma will be administered at a rate of 100 mL/h with close clinical monitoring. In both groups cointerventions including antibiotics, antivirals, heparin thromboprophylaxis, and immunomodulators such as steroids and tocilizumab will be allowed under the institutional protocol.

**Outcomes**

**Primary Outcomes:**

Composite outcome of mechanical ventilation and/or hospitalization longer than 14 days and/or in hospital-death

**Secondary Outcomes:**

- Clinical: days of oxygen requirement; days in mechanical ventilation; days of high-flow nasal cannula (HFNC) requirement; multiple organ dysfunction score (SOFA) (day 0, 3 and 7); days in intensive or intermediate care unit (ICU); time to respiratory failure development (Pa0_2_/FiO_2_<200 or, if arterial blood gases are not available on that day, a sustained Sa02/Fi02< 214 will be considered equivalent); length of hospitalization; 30 day-mortality.
- Microbiological: SARS-Cov2 PCR clearance in nasopharyngeal swab (days 0, 3 and 7)
- Inflammatory markers kinetics: lymphocyte count, C-reactive protein, procalcitonin, lactate dehydrogenase (LDH), D-dimer, ferritin and IL-6 evolution on days 0, 3, and 7.
- Radiologic: chest CT scan pneumonia score or portable chest X-ray progression between days 0 and 5.

**Subgroup analysis:**

- Immunological: IgG SARS-CoV2 titers progression (days 0, 3, 7) in participants receiving immediate plasma versus participants not receiving plasma.
- Neutralizing antibodies capacity on the day of enrolment.

**Safety Outcomes**

Adverse events (AE) will be defined as any unfavourable symptom, sign or test abnormality that is temporarily compatible with plasma transfusion. That includes COVID-19 symptoms that were not present at admission, complication associated to protocol issues (phlebitis, extravasation, etc.) and chronic disease worsening. Serious adverse event (SAE) will be defined as death, life-threatening adverse event, prolongation of hospitalization due to an adverse even, and any adverse event that requires an intervention. Death attributed to COVID-19 infection was not considered an SAE.

Any adverse event will be characterized by seriousness, duration, causality suspicion and intervention requirement. To establish causality the temporal relationship, previous pattern of transfusion-related AEs and exclusion of other causes will be reviewed.

**Follow-up**

The follow-up for efficacy outcomes will conclude at hospital discharge. Safety outcomes will be reported until 30 days after hospital discharge.

**Data collection and management**

Study follow-up and the variables that will be collected are described below.

Baseline:

Demographics, comorbidities, medications, sequential organ failure assessment (SOFA) score [31], days of COVID-19 since symptoms initiation, characteristics of COVID-19 symptoms at debut and at hospitalization, date of hospitalization.

Hemodynamics: heart rate, systolic and diastolic blood pressure, temperature, peripheral arterial 02 saturation, respiratory rate, arterial blood gases.

Evolution:

SOFA criteria daily until day 30 or discharge

Temperature daily until day 30 or discharge

Hemodynamics daily until day 30 or discharge

Respiratory function daily until day 30 or discharge: oxygen requirements, type of support, daily SaO2/FiO2, PaO2/FiO2 on day 0, 3, 7, 14 and 21.

Register of vasoactive drugs until day 30 or discharge

Register of antibiotics, antivirals until day 30 or discharge

Register of steroids and other immunomodulators until day 30 or discharge

Register of anticoagulants and thromboprophylaxis until day 30 or discharge

All-cause mortality at hospital discharge, and at 30 days

Cause of death.

Laboratory:

Total lymphocyte count; C-reactive protein, procalcitonin, LDH, D-dimer, troponin, pro-BNP, ferritin and IL6 evolution on days 0, 3 and 7.

SARS-Cov2 PCR in nasopharyngeal swab and in plasma on days 0, 3 and 7.

IgG SARS-CoV-2 titers on days 0, 3 and 7.

SARS-CoV-2 neutralizing antibodies capacity on day 0.

Imaging:

High-resolution chest CT scan at enrolment (day 0), and at day 5. If the patient has a contraindication or is unable to undergo a CT scan, a portable chest X-ray will replace it. For CT analysis and interpretation we will use three previously validated imaging scores for COVID pneumonia severity^6–9^. For the radiologic analysis when a chest CT scan cannot be performed and is replaced by a portable chest X-ray, we will add a blinded thoracic radiologist expert evaluation for improvement, stability or worsening of lung infiltrates.

Quality control:

Several procedures will assure data quality, including (1) all investigators will have certified good clinical practices training, (2) data collection will be done daily exclusively by three trained clinical research nurses; (3) a data manager will check daily data provided in the CRFs for missing data, plausible, possible or non-permitted value ranges, and logic checks. (4) any of the inconsistencies or missing data will be notified to the clinical project manager and asked to correct them before entering data in the final electronic database; (5) the principal investigator will review detailed weekly reports on screening, enrollment, follow-up, inconsistencies and completeness of data.

**Sample Size**

Sample size was calculated with a power of 80% and a statistical significance of 5% for an expected primary outcome in 54.8% of the patients in the control group and 20% of patients in the intervention group (absolute risk reduction of 35%) based on a previous immune plasma experience in AH1N1 influenza^10^. The final calculated sample size was of 29 individuals per group (total n = 58)

**Statistical Analysis Plan**

Continuous variables will be shown as mean and standard deviation for normally data or median and interquartile range elsewhere. Categorical variables will be shown as the number of cases and percentages

Primary outcome

We will assess the effect of early plasma compared to delayed plasma on the primary binary outcomes through chi-squared tests or Fisher’s exact test when appropriate, and odds ratios will be presented together with 95% CI and p-values. In addition, as an exploratory analysis we will adjust for age and SOFA score at enrolment, and clinically significant baseline variables if they result imbalanced between both study arms, as fixed (individual-level) effects, using logistic regression. Values of p<0.05 (2 tails) will be considered.

Secondary outcomes

We will assess the effect of early plasma compared to delayed plasma on the secondary binary variables through chi-squared tests or Fisher’s exact test when appropriate. For continuous variables with normal distribution we will use the t-test, and for continuous variables with non-normal distribution the Wilcoxon-rank-sum test or Mann-Whitney U test for comparisons. Additionally, we will analyze binary variables using logistic regression. Numerical variables will be examined using generalized linear models with log link function and gamma family function. Treatment effects, crude and adjusted, will be presented as exponentiated coefficients, i.e. odds ratios, hazard ratios or incidence rate ratios, respectively and treatment effect estimates will be presented with 95% CI.

To test difference between Kaplan-Meier estimates in survival analysis, we will use the Log-Rank test and/or Cox proportional hazard models for time-to event variables.

For the analysis of paired continuous variables, we will use Wilcoxon matched-pairs signed-rank test.

Statistical significance will be defined using a 2-sided significance level of α = .05.

Immune response subgroup analysis

A pre-planned analysis of baseline neutralizing antibodies titers and anti-SARS-CoV-2 IgG titers progression (on days 0, 3, 7), is planned for participants from the early plasma group and for the subset of participants from the deferred plasma group who will not have yet received plasma on those scheduled days. Groups will be compared through Chi-squared tests or Fisher’s exact test when appropriate.

Statistical significance will be defined using a 2-sided significance level of α = .05.

The statistical analysis will be performed by an investigator who was blind to the study group allocation.

**Handling of missing data and dropouts**

Patient with incomplete follow-up or protocol violations, regardless of the reason, will be included in the primary outcome analysis by intention-to-treat (ITT) principle.

**Interim analysis**

After the first 4 weeks from the beginning of the trial or 50% of enrolment achieved, an interim analysis will be conducted to evaluate enrolment difficulties, safety data, data quality and eventual amendments in any relevant methodological outcome.

**Ethics and dissemination**

This study was approved by the Institutional Review Board of the Pontificia Universidad Católica de Chile. Plasma donors are volunteers and will be asymptomatic for 28 days at least, with negative SARS-CoV-2 polymerase chain reaction (PCR) both in nasal swab and blood. Informed consent will be solicited to each patient, or their representative in cases in which the patient cannot consent. The patient or their representative can withdraw their consent whenever they choose to. Patient data will be anonymized and entered in a database only accessible to the study team.

**References**

1. Ji D, Zhang D, Xu J, et al. Prediction for Progression Risk in Patients with COVID-19 Pneumonia: the CALL Score. *Clin Infect Dis*. 2020;0954162(478):1-4. doi:10.1093/cid/ciaa414

2. Ministerio de Salud de Chile. Estandares para la obtención de componentes sanguineos y gestión de stock. 2013:1-63.

3. Singanayagam A, Patel M, Charlett A, et al. Duration of infectiousness and correlation with RT-PCR cycle threshold values in cases of COVID-19, England, January to May 2020. *Euro Surveill*. 2020;25(32):1. doi:10.2807/1560-7917.ES.2020.25.32.2001483

4. Schmidt F, Weisblum Y, Muecksch F, et al. Measuring SARS-CoV-2 neutralizing antibody activity using pseudotyped and chimeric viruses. *J Exp Med*. 2020;217(11). doi:10.1084/jem.20201181

5. Sarzotti-Kelsoe M, Bailer RT, Turk E, et al. Optimization and validation of the TZM-bl assay for standardized assessments of neutralizing antibodies against HIV-1. *J Immunol Methods*. 2014;409:131-146. doi:10.1016/j.jim.2013.11.022

6. Pan F, Ye T, Sun P, et al. Time course of lung changes at chest CT during recovery from Coronavirus disease 2019 (COVID-19). *Radiology*. 2020;295(3):715-721. doi:10.1148/radiol.2020200370

7. Zhou S, Zhu T, Wang Y, Xia LM. Imaging features and evolution on CT in 100 COVID-19 pneumonia patients in Wuhan, China. *Eur Radiol*. 2020:1. doi:10.1007/s00330-020-06879-6

8. Yang R, Li X, Liu H, et al. Chest CT Severity Score: An Imaging Tool for Assessing Severe COVID-19. *Radiol Cardiothorac Imaging*. 2020;2(2):e200047. doi:10.1148/ryct.2020200047

9. Pan F, Zheng C, Ye T, et al. Different computed tomography patterns of Coronavirus Disease 2019 (COVID-19) between survivors and non-survivors. *Sci Rep*. 2020;10(1). doi:10.1038/s41598-020-68057-4

10. Hung IFN, To KKW, Lee CK, et al. Convalescent plasma treatment reduced mortality in patients with severe pandemic influenza A (H1N1) 2009 virus infection. Clin Infect Dis. 2011;52:447–56. doi: 10.1093/cid/ciq106

**.**
