# Supplementary material for: Early versus deferred anti-SARS-CoV-2 convalescent plasma in patients admitted for COVID-19: A randomized phase II clinical trial
Source: PLoS Med. 2021 Mar 3;18(3):e1003415. doi: 10.1371/journal.pmed.1003415 (PMC7929568; doi:10.1371/journal.pmed.1003415)
Supplement: Attachment — Submitted filename: RESPONSE TO REVIEWERS.docx [file pmed.1003415.s006.docx]

The authors would like to sincerely thank the Editors and Reviewers for their thorough review of this manuscript. Their revisions contributed to significantly improve the correct reporting, analysis as well as to the discussion of this study.

**Requests from the editors:**

**Editor:** *In the Abstract "methods and findings" section, please make sure all sentences (eg the below) are full sentences, ie with a verb, rather than sentence fragments: "Open-label, single-center, randomized clinical trial performed in an academic center in Santiago, Chile, from May 10, 2020, to July 18, 2020, with final follow-up August 17, 2020".

**R:** All sentences were modified as requested (page 2: lines 45, 52, 60)

**Editor:** *In multiple places in the manuscript (see also below, and highlighted by the reviewers), the authors should rephrase the main conclusions so that it's clear they are not claiming their study has shown lack of benefit but rather failed to find evidence of benefit, given the lack of power on primary outcome: eg Abstract, "We found no benefit in the primary outcome (32.1% vs 33.3%, OR 0.95, 95% CI 0.32-2.84, p>0.99) in the early versus deferred CP group."

**R:** All main conclusions were rephrased in the Abstract, Results and Discussion to make clearer that the study was not able to show benefit rather than demonstrate no benefit. Please see changes in: Abstract results (page 2, line 56), Abstract conclusions (Page 3: line 65), in the Discussion (page 20, line 416-417, line 424; page 21 line 428-429; page 22 line 452), and in the Conclusion (page 23, lines 484-485).

**Editor:** *In the last sentence of the Abstract Methods and Findings section, please include a note summarising key limitation(s) of the study's methodology.

**R:** A sentence was added in the Abstract Methods and Findings Section indicating that the small sample size is this study key limitation (page 3, lines 62-63).

**Editor:** *Per the note above about power issues, the following statement in the abstract should be rephrased accordingly - "Immediate addition of CP therapy in early stages of COVID-19 -compared to its use only in case of patient deterioration- did not confer benefits in mortality" - this would be better read as saying "We failed to find evidence of benefit..."

**R:** The Abstract conclusion was rephrased as requested (page 3, lines 65-66).

**Editor:** *At this stage, we ask that you include a short, non-technical Author Summary of your research to make findings accessible to a wide audience that includes both scientists and non-scientists. The Author Summary should immediately follow the Abstract in your revised manuscript. This text is subject to editorial change and should be distinct from the scientific abstract. Please see our author guidelines for more information: <https://journals.plos.org/plosmedicine/s/revising-your-manuscript#loc-author-summary>

**R:** A short (378 words), non-technical author summary was added as requested (pages 3-4).

**Editor:** *In the Introduction, in the following sentence, "contagions" would be better phrased as "infections" - "The SARS-CoV-2 pandemic has left over 24 million contagions and 833,000 deaths by August 29, 2020[1]"

**R:** The word “contagions” was replaced by “infections” (page 5, line 117)

**Editor:** *In the Introduction, can you say anything to give context about the types of settings the cited case series pertain to? Eg, high, middle, low-income countries, any specific considerations about the eligibility for these series (inclusion/exclusion criteria - adults, children, specific vulnerabilities, population-representative cohorts etc?) - "Different case-series have shown an intensive care unit (ICU) admission rate between 5 and 16%, and a case fatality rate near 1-4%"

**R:** The Introduction was modified to better describe the epidemiological information from early COVID cohort and case series (page 5, lines 120-123).

**Editor:** *In the Methods section, can you add a sentence in an appropriate location noting that the trial was reported per the CONSORT guidelines and add a citation there for the CONSORT instrument?

**R:** A sentence indicating that Consort Guidelines for Reporting Randomized Controlled trials were followed was added as well as the appropriate reference (page 12, line 276-277).

**Editor:** *Per the reviewer's comment, in the Methods section where you detail power considerations, can you add a note saying whether this was an a priori or post-hoc power calculation? Many thanks - "Sample size was calculated with a power of 80% and a statistical significance of 5% for an absolute risk reduction of 35% in the primary outcome"

**R:** Our calculation of the sample size was done a priori; this information was presently included in the Methods section (page 11, line 253). Given that there was insufficient information to estimate the effect size of the intervention at the time our study was planned, our research team decided to use previous and related findings on the subject and similar research questions (as one of the recommended strategies for these scenarios) such as convalescent plasma administration in the early stage of AH1N1 influenza. We recognize that the sample size estimation could also have been obtained through a pilot study, but we decided not to use this option because it would take more time, and due to the severity of the pandemic and the uncertainty of its duration, we were felt compelled to get results as soon as possible.

**Editor:** *In the Discussion, although in some places the conclusions are framed as a failure to find benefit, in other places it's stated as a finding of lack of benefit, eg the following - "The lack of clinical benefit from convalescent plasma therapy in these patients may be explained by several reasons" - in each case this needs to be reframed so the discussion is focussed around the wide CI's for the effects on main outcomes and therefore with the evidence from this trial being consistent with a range of possible effects (including possible benefit).
*The above point also pertains to the following statement, and perhaps others in the discussion too - "Secondly, limited efficacy may be due to a late administration in the course of the disease..."

**R:** The Discussion was reframed at each of the sentences where lack of benefit was previously directly or indirectly mentioned, to make clear the uncertainty of the study findings given the small sample size. We have also directly mentioned in the study limitations that the study may have been underpowered and that given the wide CI’s for our main outcomes we cannot exclude that a larger clinical trial may end up showing clinical benefits from convalescent plasma therapy (see page 20, lines 416-417 and line 424; page 21 line 429; page 22 lines 452-453 and 468-471)

**Editor:** *Finally, the final take-home message in the Discussion (below) will also have to be moderated, given the above point - "In conclusion, convalescent plasma transfusion in patients hospitalized in the early stage of COVID-19, compared to giving plasma only at clinical deterioration, did not improve clinical outcomes.."

**R:** The conclusion of the Discussion was rephrased accordingly (page 23: lines 483-486).

-----------------------------------------------------------

**Comments from the reviewers:**

**Reviewer #1:**

This small RCT on convP for COVID has as the unique aspect that patients were treated within 7d after symptom onset. Although the study is clearly too small to draw definite conclusions on efficacy, it gives some relevant insights into antibody kinetics after transfusion.

**Reviewer:** line 77:  The authors should briefly mention the evident positive impact of dexam on mortality and the shorter time to recovery with remdesivir.

**R**: We have now included a mention to the findings of both dexamethasone and remdesivir main clinical trials (page 5, lines 126-128).

**Reviewer:** Donors were screened based on an anti-SARS-CoV-2 (S1) IgG titers 1:400 (ELISA Euroimmun®). It is not entirely clear to me how this 1:400 relates to OD ratio (the official measure of the Euroimmun test). To get an indirect idea of the PRNT50 titers in donor plasma the authors should mention the euroimmun OD ratio of the donor serum rather than the titer as the OD ratio correlates well with the SARS2 plaque reduction neutralization essay (see <https://www.nature.com/articles/s41467-020-17317-y/figures/1>).

**R**: We are aware that the Euroimmun® ELISA is not a quantitative assay. However, when we started our study, neither quantitative ELISAs neither PRNT50 assays were available in our facilities. Early reports had shown that IgG end-titers were correlated to neutralizing antibody titers (Suthar et al. doi.org/10.1016/j.xcrm.2020.100040).

Given that for the Euroimmun® ELISA, the ratio for a positive assay (ratio of sample OD/calibrator ≥ 1.1) is determined -as per the provider- with a baseline dilution of 1:100 (10uL of serum in 1.0mL), we decided to further semi-quantify the IgG in donor plasma with an additional 1/400 dilution (considering again a ratio ≥1.1 as a positive result for that new dilution), and established that 1/400 cut-off as the requirement for our plasma donors.

Thus, we firstly screened donors with the standard Euroimmun® ELISA test and additionally - for those donors resulting with a positive result at the standard basal dilution-, we went into a new fourth-fold dilution (considering again a ratio ≥1.1 as a positive result for that dilution) and established that the 1/400 cut-off as the requirement for plasma donors.

Later, we have the opportunity to standardize the PNRT50 assays and measured retrospectively the nABs to 18 infused plasmas.

We have now explained in detail in the Methods section the IgG serology titles process (page 8 lines 185-189) and we have also included the following sentence to address this suggestion:

“The median SARS-CoV-2 IgG OD ratio at the standard basal dilution (1:100 dilution) for the donors (n=41) whose plasma was administered to the patients in this clinical trial was 5.73 (IQR 4.73-6.51). A total of 18 (44%) of these infused plasmas had their neutralizing capacity determined, and the median titer of neutralizing antibodies (NAb) ID50 was 449 (range: 147 - 5610).” (page 8, lines 191-194)

**Reviewer:** The authors should also mention what percentage of the donors were rejected based on this 1/400 cut-off. e.g Did this cutoff lead to the use of the 10% highest titers of all donors tested or was it the top 5 or 25%?

**R**: We have revised this information and added a new sentence as follow:

"From a total of 232 plasma donors with baseline positive IgG SARS-COV-2 detection,129 candidates (55.6%) achieved the positive cut off at the 1/400 dilution.” (page 8, lines 189-191)

**Reviewer:** Line 163: At 1/101 should probably be 1/10 or 1/100. Please correct

**R:** Thank you for this remark. This was a typing error, so we change to:

“…diluted at 1:100”

**Reviewer:** The power calculation cannot be correct in my opinion. Every 35% reduction study has a sample size of hundreds of patients unless one would anticipate that the endpoint occurs in the majority of the controls (which is not the case). Also, a sample size calculation should be completely reproducible by an independent statistician who reads the paper. Therefore, it should mention the exact assumptions and methods used: What was the expected incidence of the prim endpoint in the control arm and the intervention arm.  If this was a post-hoc power analysis, it should be removed and the authors could state instead that no power calculation was performed.

**R:** We agree with the reviewer's impression about the calculation of the sample size, but we would like to give a supplementary explanation to justify our estimate. In the reference study used to estimate the sample size, the outcome occurred in 54.8% of the patients in the control group and 20% in the intervened group (absolute risk reduction of 35% in the primary outcome). Given that the event was highly frequent in the control group, we included patients with a similar high-risk profile (a CALL score higher than 9 predicts almost 50% risk of progression into severe COVID-19). Despite this, in our clinical trial the outcome occurred with a lower frequency than expected, and we believe that one of the explanations was the use of corticosteroids, whose usefulness was not known at the time of our research was planned.

We have completed the requested information for the complete sample size calculation reproducibility. In addition to the expected absolute risk reduction, we also have detailed what was the expected incidence for the main outcome in the intervened and in the control groups (page 11, lines 253-255).

**Reviewer:** The study had one primary endpoint (composite of death, mech ventil or >14d hospital stay). So only one statistic should have been predefined. Instead, the authors write that the primary binary outcomes were assessed through chi-squared tests or Fisher's exact test when appropriate, and odds ratios (ORs) are presented together with 95% CI and p-values. That is confusing. Which test was used for the primary endpoint?

**R:** The sentence was corrected to clarify that the primary outcome was assessed through Fisher’s exact test, and that the odds ratio (OR) is also presented together with 95% CI and p-value (page 11, line 258-259).

**Reviewer:** Given the small sample size, the number of analyses endpoints is extremely high. I suggest to move most of the secondary endpoints to a online supplement. Even the analysis on the different subgroups of the compos prim endpoint are not very relevant and could be removed or moved to online supplement. The same can be said for the baseline characteristics table.

**R:** We have now moved and renamed Table 3 (laboratory outcomes), Table 4 (radiological changes) as well as Figure 4 (Chest CT COVID-19 pneumonia severity scores) to the online supplementary material as S2 Table, S3 Table and S1 Figure, respectively. We prefer to keep Table 1 (main characteristics of study participants) in the main manuscript, as this is an important table for clinical trials as per CONSORT reporting guidelines.

**Reviewer:** Fig3. The positive/negative PCR result analysis is likely to be  a very insensitive method because even in a patient who has fully recovered from COVID, PCR can remain positive for weeks to months. A better analysis would be to look at the change in cycle threshold values from d1 to day 3 or from d1 to day 7 and compare this between CP and controls (excluding those from the control group who received rescue Cplasma before day 3). I urge the authors do do this as a post hoc analysis and describe the results (and mention that it was a post-hoc analysis).

**R:** We initially presented the PCR results as positive/negative/indeterminate because of variability associated to the sampling. However, we incorporated the reviewer’s suggestion and did this new analysis looking at the change in cycle threshold from Day 0 to Day 3 and Day 0 to Day 7 in both study arms (excluding patients from the deferred plasma group that received CP). Again, we found no significant difference between both study arms. This is shown in the Results section (page 16-17, lines 335-338) and as part of the Figure 3 (as a Figure 3b).

**Reviewer:** The discussion should very clearly mention that the study can in no way conclude that plasma therapy is ineffective given the small sample size (and the sample size calculation that I assume is incorrect). So the statement "it is possible that the study underpowered" should be removed and be clear about the lack of power to detect a clinically relevant effect size.

**R**: We have rephrased the main study limitation regarding the relevance of the small sample size and modified the conclusions accordingly in the Abstract (page 3: lines 62-63) and in the Discussion section (page 22: lines 468-471).

**Reviewer:** However, the study does provide  relevant information about the knowledge gaps we still have:  - Why are not all patients antibody positive on day 3 after  the plasma transfusion? Is it because the dosing is inadequate ? Have the antibodies been consumed quickly by attaching to the virus and is a much higher dose or plasma with much higher titers needed (e.g. some studies are using 4 units over 2 days). Has the window of opportunity been past when patients are sick enough to enter the the hospital due to a overwhelming inflammatory response, even if sick for <8 days?

**R:** These are very interesting observations, we have added a comment on the lack of seropositivity in about a third of plasma-infused patients on Day 3, and how this finding may relate with a possible lack of efficacy (insufficient dose of infused plasma as a relevant hypothesis) (page 20: lines 424-426). The comment on the window of opportunity was already addressed on page 21 line 444-446 and page 23, lines 474-477.

**Reviewer:** A limitation that should be mentioned is that typically +- 75% of the patients will have passed the 7d symptoms window when they are admitted to the hospital. Another limitation that should be mentioned is the lack of any dose finding data on conval plasma in healthy volunteers and the fact that the antibodies in 400 ml of plasma given to an adult patient will be diluted in +-6 times this volume, let alone the consumption of antibodies that leave the blood compartment and/or bind the virus.

**R:** A paragraph was added in the Discussion section regarding time from symptoms onset to hospital admission as a barrier for early plasma administration (page 23, lines 474-477). Also, we added a sentence in the study limitations to further comment on the lack of knowledge regarding the plasma doses required to effectively neutralize the virus *in vivo* (page 22: lines 465-469)

**Reviewer:** The study should give some short recommendations on how further antibody based therapies (plasma, monoclonal highly neutral antibody therapy) should be studied. Some suggestions are : Focus on high risk patients before admission. First establish what dose is appropriate in H volunteers. Multicenter large trials etc.

**R**: We added to the Discussion section a proposal on how convalescent plasma-based therapy should be further studied, including as critical steps timing and dosage (still not defined) (page 23: lines 477-482)

-----------------------------------------------------------

**Reviewer #2:**

This was an open, labelled, single-center, randomized clinical trial conducted at an academic center in Santiago, Chile. It focused on the application of convalescent plasma in COVID-19 patients. The 58 patients included in the study were randomly divided into an early plasma group and a delayed plasma group. The authors found that there was no significant difference in the main efficacy of the early CP group and the delayed CP group. The author believed that compared to using it only when the patient's condition deteriorates, the immediate addition of CP treatment in the early stage of COVID-19 did not bring benefits in terms of mortality, length of hospitalization, or mechanical ventilation needs. The clinical research is very rigorous in design and development. However, there are several concerns in the following:

**1.** Different cellular and humoral responses are generated in mild or severe COVID-19 cases, and it has been reported that a specific cellular response can be detected early in the course of non-severe COVID-19. So what explains the cellular and humoral responses of the patients included in this study? Why not perform subgroup analysis of mild and severe covid-19 patients?

**R:** We thank the reviewer for this comment; the study of the cellular immune response is indeed very interesting, but it was not within our study scope or objective. Regarding the proposal for mild and severe COVID-19 cases subgroup analysis, we believe it would not be appropriate given the small sample size and also because our inclusion criteria excluded patients with severe COVID-19 (patients with PaO2/FiO2 <200).

**2.** Additional standard treatment was allowed in both arms. What are the main additional standard treatments? For example, does the use of steroids interfere with the conclusions reached?

**R:** Additional standard treatment during hospitalization is extensively detailed in Table 1. The proportion of patients that were treated with steroids does not differ significantly between both study arms, as shown in Table 1 (82.1% vs 66.7%, p=0.23). In the table, the numbers of patients that received steroids were corrected as we found a small error of transcription from the database, but statical significance did not change.

3. In previous studies, it has been observed that different levels of IgG plasma have a certain impact on patient mortality, and even unadjusted IgG has a dose-response relationship with 30-day mortality. How to ensure the consistency of convalescent plasma IgG collected from volunteers?

**R:** In this study, the screening process required for donors to have an anti-SARS-CoV-2 (S1) IgG titers of at least 1:400. Beyond that, as mentioned in the Discussion (page 22, lines 465-466), we did not select donors with the highest NAbs or IgG titers. Nonetheless, baseline IgG levels in all donors (n=28) whose plasma was given to the early plasma arm versus IgG level in all donors (n=13) whose plasma was administered to the deferred plasma arm, were not different (median IgG OD ratio (at standard baseline 1:100 dilution) of 5.77 and 5.73, respectively, p=0.81). This information was added in Results on page 18, line 361-364.

**4.** Different patients have different degrees of progress in the course of the disease. Some elderly people only show real COVID-19 symptoms in the later stages of the disease, and some people have asymptomatic infection.

**R:** The reviewer is right; the range of manifestations of SARS-CoV-2 infection spans from asymptomatic infections to severe disease. We have acknowledged in the Discussion that some of the patients (particularly the older) may have been unaware of “true” COVID-19 symptoms onset (page 21: lines 444-446).

**5.** The progression of COVID-19 pneumonia CT severity score from baseline to day 5 in the delayed treatment group was higher than that in the early plasma group. Perhaps you should continue to study and increase the number of patients enrolled.

**R:** The finding of higher progression of CT severity score in the Deferred plasma group (S1 Figure) was not sustained when including in the analysis the patients that were too sick to undergo a CT scan and that had to had a portable chest X-ray instead, as shown in S3 Table.
-----------------------------------------------------------

**Reviewer #3:**

Balcells et al describes a randomized trial of COVID-19 convalescent plasma (CCP) in COVID-19 infection comparing early and late administration of CCP.  A composite endpoint as well as individual secondary outcomes were examined.  The authors failed to identify any differences in primary composite outcome and a number of secondary outcomes between high risk of progression patients receiving CCP early and those receiving it only upon progression according to predefined criteria.

Overall, the paper is well written and represents important new information concerning the use of CCP in the ongoing pandemic.  I have one major concern which could influence the paper as well as a some minor concerns.

Major Concern
1) On page 5, lines 121 through 126 the COVID convalescent plasma is described including the testing performed to determine whether antibodies to SARS-CoV-2 were present and the titer of the units.  It is indicated that the Euroimmun ELISA was used to test the units and that the IgG titers were >1:400.  The Euroimmun ELISA, however, is not a qualitative assay capable of determining the titer.  Additional information and detail is needed to describe how the titer was determined utilizing this test.  For example, in the Expanded Access Protocol recently described from the United States, the Ortho VITROS SARS-CoV-2 IgG assay was compared to the Broad PRNT neutralizing assay to determine the S/C ratio corresponding with the various titers reported.  presumably something similar was done.  This should be described as the titer of the units could conceivably determine the efficacy of the plasma and therefore the outcomes of the study.  Latter it is mentioned that a subset of units
were tested to determine the titer of nAb by the authors.

**R**: As explained for Reviewer 1, we are aware that the Euroimmun® ELISA is not a quantitative assay.

However, when we started our study, neither quantitative ELISAs neither PRNT50 assays were available in our facilities. Early reports had shown that IgG end-titers were correlated to neutralizing antibody titers (Suthar et al. doi.org/10.1016/j.xcrm.2020.100040).

Given that for the Euroimmun® ELISA, the ratio for a positive assay (ratio of sample OD/calibrator ≥ 1.1) is determined -as per the provider- with a baseline dilution of 1:100 (10uL of serum in 1.0mL), we decided to further semi-quantify the IgG in donor plasma with an additional 1/400 dilution, (considering again a ratio ≥1.1 as a positive result for that new dilution), and established that 1/400 cut-off as the requirement for our plasma donors.

Thus, we firstly screened donors with the standard Euroimmun® ELISA test and additionally - for those donors resulting with a positive result at the standard basal dilution-, we went into a new fourth-fold dilution (considering again a ratio ≥1.1 as a positive result for that dilution) and established that the 1/400 cut-off as the requirement for plasma donors.

Later, we have the opportunity to standardize the PNRT50 assays and measured retrospectively the nABs to 18 infused plasmas.

We have now explained in detail in the Methods section the IgG serology titles process (page 8 lines 185-189) and we have also included the following sentence to address this suggestion:

“The median SARS-CoV-2 IgG OD ratio at the standard basal dilution (1:100 dilution) for the donors (n=41) whose plasma was administered to the patients in this clinical trial was 5.73 (IQR 4.73-6.51). A total of 18 (44%) of these infused plasmas had their neutralizing capacity determined, and the median titer of neutralizing antibodies (NAb) ID50 was 449 (range: 147 - 5610).” (page 8, lines 191-194)

**2)** On page 17, lines 314 through 316 - The authors describe possible TRALI reactions.  The overall reaction rate is much higher than that reported In the Expanded Access Program which has reported on 20,000 patients receiving CCP.  Were the donors providing the CCP either male donors, female donors who had never been pregnant, or were the donors screened for the presence of anti-HLA antibodies?  This is not stated in the initial description of the CCP.  Please indicate whether or not such TRALI risk reduction measures were implemented both in your description of the plasma product as well as in your safety results.

**R:** We have now included the donors characteristics in the “convalescent plasma donation protocol“ section of Patients and Methods section. Donors were males and females who have never been pregnant, or female donors who have been tested for HLA antibodies (Pag 7, lines 178-179).

**3)** On page 17, lines 316 through 321 the authors describe profound thrombocytopenia following transfusion of CCP in a single patient. They mention that testing for antiplatelet antibodies was negative. Please clarify whether this testing was performed in the recipient or in the donor. If in the recipient, reports of passive alloimmune thrombocytopenia (PAT) have appeared in the literature and in many of these, antibodies were not detected in the recipient despite being present in the transfused blood product. Absence of antiplatelet antibodies in the recipient may not exclude this entity.

**R:** After this reviewer comment, we have come back and tested for antiplatelet antibodies the infused plasma (we had frozen aliquot preserved for all infused plasmas) and they resulted negative. We have now added that information in the Safety results (Pag 20: lines 409-410).

Minor
**1)** Page 16, line 286 - The phrase "plasma receptors" is rather awkward and not correct English.  I would suggest rewriting as "...seroconversion rates between those who received plasma and those who received no plasma at day 3..."

**R:** Many thanks. The phrase was corrected.

-----------------------------------------------------------

**Reviewer #4:**

This is a useful RCT on the efficacy of early Anti-SARS-CoV-2 Convalescent Plasma treatment vs late plasma treatment as needed in Patients Admitted for COVID-19. The statistical methods and analyses and presentation of the results (tables and figures) are mostly adequate. However, there are some major issues needing attention especially on the sample size.

**1)** Sample size. The sample size of 58 was calculated based on the assumption of an absolute risk reduction of 35% in the primary outcome. This is a huge effect size needed to be carefully justified. The authors used the paper on "Hyperimmune IV Immunoglobulin Treatment" as the reference for this 35% risk reduction, which however could not be found in the paper. The paper itself also acknowledged lack of statistical power due to small sample size, and the disease, treatment arms and primary outcome are very different from those in this paper. Thinking about the results of this study, the primary outcomes for both arms are only 32% to 33%, then an absolute risk reduction of 35% is basically not realistic and unfounded. Therefore the study is seriously underpowered due to unrealistically estimated large effect size which led to all the non-significant results with very wide 95% confidence intervals.

**R**: We are grateful for the reviewer's comments and we understand the comments regarding our estimation of the sample size. Scenarios such as the COVID-19 pandemic have generated many challenges, and one of them is the design of RCTs. At the time of our study planning (April 2020), there was not enough information about the effect of CP in COVID-19 to make a precise estimate of the sample size. In view of this, our research team decided to access one of the recommended strategies for these scenarios: use previous and related findings on the topic and on similar research questions. For this reason, we used the study on the effect of convalescent plasma in patients with H1N1 infection, another pandemic respiratory disease. When reading this reviewer comments we were surprised he mentioned that we were citing an "Hyperimmune IV Immunoglobulin Treatment" paper and even more that he could not found the numbers for sample size calculation in the paper. We only realized at that moment that with the reference manager we had mistakenly uploaded a different paper from the same authors (exactly the same authors in order and very similar title).

**The correct reference is:** “Hung IFN, To, KKW, Lee CK, et al. Convalescent Plasma Treatment Reduced Mortality in Patients With Severe Pandemic Influenza A (H1N1) 2009 Virus Infection. Clinical Infectious Diseases 2011;52(4):447–456”

Instead of: “Hung IFN, To KKW, Lee CK, et al. Hyperimmune IV immunoglobulin treatment: A multicenter double-blind randomized controlled trial for patients with severe 2009 influenza A(H1N1) infection. Chest; 2013;144: 464–473.”

**In the correct reference (Hung IFN et al. CID, 2011) the editors and reviewers will find the expected outcomes and absolute risk reductions from convalescent plasma that were used to calculate our study sample size**.

We are sorry for this mistake. Old reference nº26 was now corrected (now as Ref nº28).

Still, we understand that presently an absolute risk reduction of 35% could be considered unrealistic; however, there was no information about the effect of CP in patients at high-risk for developing the outcome when planning the design of our study. Besides, we did not expect a low percentage of patients with the primary outcome in the control group. We believe that this could be due to the use of co-interventions that we now know reduce COVID-19 mortality such as corticosteroids. We have anyway mentioned in Abstract and in the Discussion our small sample size as a study limitation.

**2)** Study design. As we are still not clear whether the convalescent plasma is useful/effective at all in treating Covid19, we would think that a RCT on plasma vs no plasma would be the priority but why on early plasma treatment vs later plasma treatment as needed? It's intuitive that if there is any effect on plasma vs no plasma, then the effect on early vs late plasma will be smaller, but an absolute risk reduction of 35%? We know that Phase II trials typically tend to find a larger effect size of over 10% but 35% seems too big and not justified.

**R:** When this study was designed (April 2020), there were so many expectations on convalescent plasma as a possible effective therapy for COVID-19 (and no other known effective alternative therapies) that we considered it would be almost impossible for patients and their families to accept - in the peak of the pandemic - being enrolled in a study to receive nothing or a placebo as treatment for a highly deadly and feared disease.

**3)** Discussion. There was a light touch on the sample size in the limitation section however the small sample size issue/impact needs to be seriously and comprehensively addressed in the limitation. As this is a Phase II trial, the authors need to focus on the purpose of the phase II trials, really need to tone down the claims as seriously lacks of stats power due to small sample size, should instead focus on from the results whether a further confirmatory phase III is warranted.

**R:** As already mentioned in the responses to the editors and the other reviewers, we have now carefully addressed the small sample size as an important limitation and tone down the conclusions from our study findings.

**4)** Table 2 is a key table. Need to mention in details in the footnote a) what were adjusted in the adjusted analyses? 2) what stats models were used to give all the ORs and IRRs?

**R:** The following sentence was added in Table 2 footnote: “*Adjusted ORs were estimated from a logistic regression model and IRRs were estimated using zero inflated negative binomial model. Estimates were adjusted by age and SOFA score at enrolment.*”

-----------------------------------------------------------

**Reviewer #5:**

In this article, Balcells and colleagues present the results of their study titled "Early anti-SARS-CoV-2 Convalescent Plasma in Patients Admitted for COVID-19: A Randomized Phase II Clinical Trial". The study design included an early and a deferred COVID-19 convalescent plasma (CCP) treatment group. Block randomization was used to assign patients with documented SARS-CoV-2 infection who were ≤7 days from symptom onset and high risk of respiratory decompensation to the early group, which received CCP on enrollment; one unit at enrollment and one unit within 24 hours) or the deferred group, which received CCP if they met the criteria for respiratory decompensation. There were 28 patients in the early group and 30 in the deferred group, from which 13 received CCP.

Based on their statistical analyses, the authors conclude that there was no statistical difference in composite (death, mechanical intubation, hospital stay) status on day 14 (primary outcome) or day 28 (secondary outcome). They also conclude there were no statistical differences in SARS-CoV-2 seroconversion or changes in inflammatory markers between the groups.
Despite the negative outcome of this study, there are numerous patient characteristics and other factors that may have influenced the analysis and conclusions. The following comments and questions are provided for the authors' consideration.

**1.      Patients:**
**a.**      Despite randomization, the early treatment group appears to have been sicker. They had lower lymphocytes, higher ferritin and D-dimers, and more severe pneumonia on CT imaging (Table 1). In addition, more patients in this group were on steroids and fewer received anti-coagulation. Did these variables affect outcome?

**R**: We are aware that baseline data showed small differences between groups in variables such as use of steroids and anticoagulation as well as the severity of pneumonia, ferritin levels and D-dimer. Although we did not find statistically significant differences between groups in these baseline covariates, we explored their potential impact on outcomes by including them as confounding variables in the multivariable regression models. We found that none of these variables had impact on the outcomes.

**b.**      The mean age of both cohorts was older than cohorts reported in other studies about which this reviewer is aware. Older patients may have less effective antibody (or adaptive) responses and more rapid disease progression. The range in age was wide in each group. Did outcomes vary as a function of age?

**R**: We agree with the reviewer that advanced age is the main risk factor for worst outcomes. Therefore, the study main outcomes were analyzed as crude results and also **adjusted for age, and severity on admission** (SOFA score). Unadjusted and adjusted ORs are shown in Table 2. Adjusted results did not differ significantly from unadjusted.

**c.**      Though not statistically significant, there were more deaths in the early group at 14 and 28 days (Table 2). Were the patients who died older? Sicker (lower lymphocytes, higher D-dimer, more severe pneumonia)? Did their antibody levels differ? More information on these patients should be provided.

**R**: As suggested by the reviewer, we revised the clinical and laboratory characteristics of the patients who died (n=7) compared to the patients that survived (n=50). We found that older age (p=0.0006) and higher SOFA score on admission (p=0.0024) were associated with subsequent risk of death (and these were in fact the two baseline variables that were a priori included in the multivariable analysis for the RCT outcomes). Regarding the laboratory parameters at enrolment, procalcitonin (p=0.02), IL6 (p=0.001), pro-BNP (p<0.0001) and troponin T (p<0.0001) were significantly higher in patients that died at follow-up than in patients that survived. Also, none of the patients that died had positive IgG antibodies on enrolment and the median IgG SARS-Cov-2 levels (baseline IgG OD ratio 0.29 vs 0.27, p=0.47). We briefly added these findings in the text (page 17, line 354-358 and page 18, lines 376-377).

**d.**      The number of patients who had SARS-CoV-2 IgG at day 3 was higher in CCP recipients, albeit not statistically significant. What were the titers? Did titers vary with age? Other patient characteristics? What were the actual titers on day 0?

**R**: On day 0, 7/26 (27%) of patients who received CP had positive SARS-CoV-2 IgG with median titers of 400 (range: 100-800), compared to 5/20 (25%) of those who did not receive early CP and had median IgG titers of 400 (range: 100-3200), a non-significant difference in titles (p=0.55). On day 3, 19/26 (73%) of patients who received CP had positive SARS-CoV-2 IgG with median titers of 400 (range: 100-3200), compared to 10/20 (50%) of those who did not receive early CP and had median IgG titers of 400 (range: 100-3200), a non-significant difference (p= 0.96).The titers did not correlate with age (r=0.03 p=0.87, Spearman Test). Two brief sentences were added to the result section to include this analysis (page 18, line 365-372)

**2.      Study design:**
**a.**      13 of 30 patients in the deferred group received CCP. Did it affect their outcomes?

**R:** Of those 13 patients that received CP in the deferred plasma group, 9 met the primary combined end point anyway (including 2 deaths), therefore CP did not prevented the primary outcome to happen. However, given the study design, this group has not a good comparator, because they received in their vast majority (12 out of 13) the plasma due to respiratory failure progression criterion (PaO2/FiO2 <200).

**b.**      SARS-CoV-2 IgG positivity and neutralizing antibody levels are provided for both groups. Are IgM, IgA, or levels of antibodies to other determinants (e.g. nucleocapsid) available?

**R**: No, we did not determine IgM or IgA, neither IgG levels of antibodies to other antigens besides S1 in this study.

**c.**      Although the study was powered based on another pandemic respiratory disease, it may have been underpowered to detect differences in clinical status or mortality for COVID-19, which can be a prolonged illness.

**R**: We have now discussed and addressed as a study limitation the small sample size in all the manuscript main sections. Regarding the time to the outcomes evaluated, all our patients were followed for the total of the in-hospital period and for a minimum of 30 days after enrollment. Outcomes in the manuscript are reported as such.

**3.      Plasma:**
**a.**      Who were the CCP donors? Did all have symptomatic illness? If symptomatic, what was their degree of illness? Were they hospitalized? On what day after symptoms resolved were donations collected (the protocol says at least 28 days after symptoms)?

**R**: Donors were volunteers that had a history of a confirmed SARS-CoV-2 infection. A total of 91% had symptomatic (COVID-19) disease: 95% had a mild disease (not hospitalized) and 5% a moderate disease (hospitalized but did not require oxygen). No donor had severe disease. Convalescent plasmas were collected between 33 to 73 days after symptoms resolved (mean of 44 days). This information was added to the convalescent plasma donation protocol description (page 7-8, line 179-182).

**b.**      It appears that neutralizing titers were only available on 18 of the 41 CCP units that were administered (line 288: "… all infused plasma samples tested (n=18) had a positive ID50 at screening dilution… with a median screening dilution of 97…"). What was the actual neutralizing titer of these units?

**R**: Thanks for your comment. Indeed, in the original version of this manuscript, we have determined the neutralizing antibodies (at screening dilution only) for 18 of the 41 CP administered. As requested by this reviewer, we have now determined the final neutralizing titers in these 18 infused plasmas, with a standardized PNRT50 assay. We have incorporated this new information in the following sentence:

“…the median titer of neutralizing antibodies (NAb) ID50 was 449 (range: 147 - 5610.)” (page 8, lines 193-194 and page 18, lines 378-379)

**c.**      The actual SARS-CoV-2 antibody titers of the CCP administered (line 124 states ≥1:400) and if available, neutralizing titers, would be helpful in assessing the possibility that CCP recipients may have received low/er titer plasma. This is underscored by the negative results of the trial in India in which CCP was deemed ineffective, but many units lacked neutralizing antibodies.

**R**: We have added the information for the baseline IgG ratio in all plasma donors as well as the neutralizing titers in a subset of 18 samples from infused plasma available for PNRT50 assay. This result, that was incorporated in the manuscript (page 8, lines 191-194) as follow:

“The median SARS-CoV-2 IgG OD ratio at the standard basal dilution (1:100) for all donors (n=41) whose plasma was administered to the patients in this clinical trial was 5.73 (IQR 4.73-6.51). Additionally, a total of 18 (44%) of these infused plasmas had their neutralizing capacity determined, and the median titer of neutralizing antibodies (NAb) ID50 was 449 (range: 147 - 5610)”.
