# Supplementary material for: Early versus deferred anti-SARS-CoV-2 convalescent plasma in patients admitted for COVID-19: A randomized phase II clinical trial
Source: PLoS Med. 2021 Mar 3;18(3):e1003415. doi: 10.1371/journal.pmed.1003415 (PMC7929568; doi:10.1371/journal.pmed.1003415)
Supplement: Attachment — Submitted filename: pmed revised paper review balcells.docx [file pmed.1003415.s007.docx]

In this revised article, Balcells and colleagues have answered the major concerns of the prior review pertaining to study design and sample size calculation. The main result of the study is that there was no difference in clinical outcome of hospitalized patients with Covid-19 randomized to early versus delayed treatment with convalescent plasma. The baseline clinical data of the cohort suggest that the patients had severe disease and were at high risk for clinical deterioration, despite being enrolled ≤ 7 days after symptom onset. Neutralizing antibody titers were measured on 18 of 41 units of convalescent plasma that were administered. There are multiple factors that may have precluded a beneficial effect of plasma in this study, including that the patients had pneumonia and evidence of inflammatory progression at enrollment, were at risk for deterioration based on disease severity and age, and the administered plasma may not have had sufficient antibody to mediate an antiviral effect. These points, which are addressed well by the authors, are important to the field as it wrestles with the challenge of establishing a possible benefit of convalescent plasma and informing clinical guidance for its use. There are a few points that should be addressed to increase clarity.

Lines 420-422: The statement that “…convalescent plasma was not associated with a higher rate of SARS-CoV-2 clearance … suggesting it does not provide a strong antiviral activity at this stage” may be true. However, the data do not support this conclusion – neutralizing titers were determined on less than half of the units administered and some of the units had neutralizing titers that were < 1:160. This statement should be modified. In addition, it would be helpful to present the actual Ct values rather than the ratios. It is not possible to determine the patients’ viral burdens from the ratios.

Lines 423-426: The lack of antibody positivity in transfused patients is curious. Does the group of patients who did not exhibit a rise in antibody level include patients who did not have antibody at enrollment? The higher neutralizing titers of patients with a longer symptom duration is consistent with other studies (see Garcia-Beltran et al. medRxiv).
