# Supplementary material for: Early versus deferred anti-SARS-CoV-2 convalescent plasma in patients admitted for COVID-19: A randomized phase II clinical trial
Source: PLoS Med. 2021 Mar 3;18(3):e1003415. doi: 10.1371/journal.pmed.1003415 (PMC7929568; doi:10.1371/journal.pmed.1003415)
Supplement: Attachment — Submitted filename: renamed_cb3ea.docx [file pmed.1003415.s008.docx]

The authors would like to thank the Editors and Reviewers once again for their thorough review of the manuscript. Their revisions contributed to significantly improve the correct reporting, analysis as well as to the discussion of this study.

**Requests from Editors:**

1.      Please address the reviewers’ final concerns, below.

2.      Please amend the title to: “Early versus deferred anti-SARS-CoV-2 convalescent plasma in patients admitted for COVID-19: A randomized phase II clinical trial”

R: The title was amended accordingly.

3.      Abstract:
a.      Line 46: Please rephrase to: “…with final follow-up until…”

R: The sentence was corrected.
b.      Line 49: Please rephrase to: “…consisted of immediate…”

R: The sentence was corrected.
c.      Please add a second limitation, eg. that NAbs were not confirmed in the donor plasma. R: This was added as a second limitation in the Abstract.
d.      Line 63: Please replace “clearly show benefits” with “show effects”
 R: The sentence was corrected.

4.      Author Summary:
a.      Overall, we would recommend shortening your Author Summary by 1-2 bullet points per section.

R: The author summary was shortened to 2-3 bullet points per section. Please let us know if this is Ok or we necessarily need to further cut-off.
b.      Line 74: Please rephrase to: “The severe acute…”

R: The sentence was rephrased.
c.      Lines 81-82: Please rephrase to: “…few randomized clinical trials have been carried out to show any clinical benefit…”

R: The sentence was rephrased.
d.      Line 83: Delete this sentence: “The administration of convalescent plasma in earlier stages of COVID-19 (before respiratory failure development) was expected to be of higher benefit.”

R: The sentence was deleted.
e.      Line 102: Delete this sentence: “No significant differences were observed in IgG SARS-CoV-2 seropositivity rate nor in IgG seroconversion rates between plasma receptors and no plasma receptors.”

R: The sentence was deleted.

5.      Please add a space between text and the citation callouts, eg.: “…COVID-19 in the US [4].”
 R: A space was added between the text and all citations callouts.
6.      Line 185: Please remove any trademark symbols

R: Trademarks symbols were removed from the manuscript as well as from S1 Text File.
7.      Table 1: Please remove the p value comparisons of baseline groups, as recommended by CONSORT.

R: p-values were removed from Table 1.

8.      Although you say in several places that the trial was "underpowered"; in fact you seem to have recruited the number of participants that you planned to, implying that you can indeed exclude a 35% or greater risk reduction in the primary outcome in this study (though the trial was not powered to detect a smaller reduction than that). Please clarify where appropriate.

R: We agree with this observation. In the Discussion section, we have replaced the sentence “*Secondly, the study was probably underpowered to detect a statistically significant difference and the wide confidence intervals that we found for the effects on main outcomes do not permit to exclude that convalescent plasma may show clinical benefit in a future larger clinical trial*.”

for “*Secondly, the study was not powered to detect a risk reduction smaller than 35% in the primary endpoint, and therefore we cannot exclude that convalescent plasma may show smaller but clinical relevant effects in a future larger clinical trial*.”

Also, in the Abstract, the limitation was changed from “*The study small sample size is a*

*limitation as this trial may have been underpowered to clearly show benefits from CP*

*administration*.” to “*The limitation of this study is the lack of statistical power to detect a smaller but clinically relevant therapeutic effect of convalescent plasma, as well as not having confirmed neutralizing antibodies in donor before plasma infusion*.”, as suggested by the Editor and Reviewer 1.

9.      Throughout, please report p values lower than 0.001 as p<0.001. Please report all other p values to 3 decimal places.

R: We have now reported all p values in the manuscript, figures and tables with three decimal places.

10.     Throughout, please use “patients” or “participants”, instead of “subjects”.

R: The word “participants” was changed to “patients” or “subjects” throughout the text.

11.     Please ensure that all statements in the Abstract are also mentioned in the main text, including this sentence: “The viral clearance rate on day 3 (26% vs 8%, p=0.20) and day 7 (38% vs 19%, p=0.37) did not differ between groups.”

R: This statement was already mentioned in Results section of the main body text (see page 16, last paragraph) as well as in Figure 3A.

12.     In Fig 1, it appears that 58 are shown to have completed the trial, whereas in your abstract you state 57. Please update the flowchart accordingly.

R: We have now updated the Fig 1, to exclude the patient that withdraw consent during the study and therefore adjusting to a total follow-up number was 57.

However, we would like to point out that although that one patient (allocated to the deferred plasma arm and never meeting criteria for receiving plasma) withdraw consent from further follow-up, we had his consent to use his information for the primary outcome and baseline epidemiological information. Therefore, that patient was included and analyzed in Table 1 and Table 2 (for primary clinical outcomes) that’s why in those two tables the total participants number is n=58 (respecting the ITT principle). In secondary analysis and supplementary tables total n is =57 as we do not have information for that patient’s radiologic and laboratory secondary outcomes that required blood sampling. Thus, alternatively, we propose to the Editor to include this patient as analyzed, maintain Fig 1 as originally submitted and correct in the Abstract to a total n=58 (no losses to follow-up), still mentioning in the Results section that 1 patient withdraw consent for further follow-up and was not included in secondary outcomes analysis.

13.     In the reference list, please delete publisher names (eg. “Elsevier Ltd”, “NLM (Medline)”, “Blackwell Publishing Inc.”) – the journal name is sufficient.

R: The reference list was revised and all publisher names were deleted.

14.     Please mark preprint references as “[Preprint]”.
 R: The reference list was revised and all preprint references are now marked as such.

15.     Please provide additional details for reference 21, (eg. a URL, DOI, or ISBN).

R: Additional details and URL were provided.

16.     When completing the CONSORT checklist, please use section and paragraph numbers, rather than page numbers.

R: Page numbers were replaced by section and paragraphs numbers in the CONSORT checklist.

17.     Please upload a version of S2 Table without tracked changes.

R: A clean version of S2 Table was uploaded.


**Comments from Reviewers:**

**Reviewer #1:** The paper improved but there remain some minor issues that should be resolved.

P3 line 62. The sentence on power of the study should be correct to state something like this because it is not "possible" but it is a fact that this study was not powered to detect smaller but still potentially clinically relevant  therapeutic effects of convalescent plasma. So please change to "The limitation of this study is the lack of statistical power to detect a smaller but clinically relevant therapeutic effect of convalescent plasma"

R: The sentence on limitations in the Abstract was changed as suggested.

P4. Please rephrase for clarity

1. Our study failed to find evidence in the primary outcome with early convalescent plasma
administration compared to its use only in case of clinical deterioration

Should be:  Our study failed to show that early convalescent plasma administration improves the outcome compared to its use only in case of clinical deterioration

R: the sentence was rephrased as suggested (see page 4, last section of Author summary)

2. This evidence could be limited by the small sample size of the study.

Should be: The small sample size of the study precludes any definite conclusions but the results are in agreement with observations from other trials on convalescent plasma for patients hospitalized with COVID19 (ref. recent BMJ paper on study in India, Medriv paper on Dutch study from Gharbharan et al. etc )
R: Thanks, the sentence was rephrased as suggested (see page 4, last section of Author summary).

P5 line 128: please change to something like this : but its effect on overall mortality remains controversial (ref Solidatory trial and ref final report of ACTT study)
R: Thanks, the sentence was rephrased as suggested (see page 5, 1^st^ paragraph).

P6 line 157: please change "but" to and imaging ….

R: Thanks, the word was replaced as suggested (see page 6, in Inclusion criteria).

P7 line 179: should be anti-HLA antibodies
R: Thanks, the word was corrected (see page 7, in Convalescent plasma donation protocol).

P8 line 189-94: please move this part to results section

R. We have moved that part (results of antibodies and nAb determination in plasma donors) to the Results section, immune response sub-section, 1^st^ paragraph.

P8 line 193 : were these 18 plasma units (not plasmata) from 18 different donors? If yes please change to "In 18 of the 40 plasma donors virus neutralizing antibody titers were measured". If no, then the number of donors not plasma units that was tested should be mentioned instead

R: Yes, these plasma units were from different donors; we rephrased the sentence for clarity (Results section, Immune response sub-section, 1^st^ paragraph).

P11b line 257. Please remove "of"

R: The word “of” was removed (Methods section, statistical analysis sub-section, 1^st^ paragraph)

P12 line 272: Only a p-value for the primary endpoint can be considered statistically significant at the  0,05 cut-off. So please add : For the primary endpoint … and add "The statistical analysis of secondary endpoints should be considered exploratory only"

R: A clarification was made in the sentence (Methods section, Statistical analysis sub-section, last paragraph).

Line 324: was higher (not resulted higher)

R: The sentence was corrected (Results section, secondary outcomes sub-section, 1^st^ paragraph).

Line325: remove "develop"
R: The sentence was corrected (Results section, secondary outcomes sub-section, 1^st^ paragraph).

Discussion;
Line 456: I don't think it was approved. It received an emergency use authorization.

R: This is right. We changed “approved” for “emergency use authorization” (Discussion section, 4^th^ paragraph)

Line 468: Secondly, the study was probably underpowered to detect a statistically significant difference and the wide confidence intervals that we found for the effects on main outcomes do not permit to exclude that convalescent plasma may show clinical benefit in a future larger clinical trial.
Please change to: Secondly, the study was only powered to detect a huge impact on the primary endpoint under study as illustrated by the wide confidence interval. Larger trials are therefore required to exclude smaller but clinical relevant effects.

R: The suggested new sentence was slightly modified to “Secondly, the study was not powered to detect a risk reduction smaller than 35% in the primary endpoint, and therefore we cannot exclude that convalescent plasma may show smaller but clinical relevant effects in a future larger clinical trial.”

474: on early stage => in an early stage …

R: The sentence was corrected (Discussion section, penultimate paragraph).

**Reviewer #3:** Thank you for addressing my concerns as well as the concerns of the other reviewers in your revision.

**Reviewer #4:** Thanks authors for their great effort to improve the manuscript. The authors have addressed my concerns/comments adequately. I am satisfied with the response and revision. No further issues needing attention.

**Reviewer #5:** In this revised article, Balcells and colleagues have answered the major concerns of the prior review pertaining to study design and sample size calculation. The main result of the study is that there was no difference in clinical outcome of hospitalized patients with Covid-19 randomized to early versus delayed treatment with convalescent plasma. The baseline clinical data of the cohort suggest that the patients had severe disease and were at high risk for clinical deterioration, despite being enrolled ≤ 7 days after symptom onset. Neutralizing antibody titers were measured on 18 of 41 units of convalescent plasma that were administered. There are multiple factors that may have precluded a beneficial effect of plasma in this study, including that the patients had pneumonia and evidence of inflammatory progression at enrollment, were at risk for deterioration based on disease severity and age, and the administered plasma may not have had sufficient antibody to mediate an antiviral effect. These points, which are addressed well by the authors, are important to the field as it wrestles with the challenge of establishing a possible benefit of convalescent plasma and informing clinical guidance for its use. There are a few points that should be addressed to increase clarity.

Lines 420-422: The statement that "…convalescent plasma was not associated with a higher rate of SARS-CoV-2 clearance … suggesting it does not provide a strong antiviral activity at this stage" may be true. However, the data do not support this conclusion - neutralizing titers were determined on less than half of the units administered and some of the units had neutralizing titers that were < 1:160. This statement should be modified. In addition, it would be helpful to present the actual Ct values rather than the ratios. It is not possible to determine the patients' viral burdens from the ratios.

R: As suggested by the reviewer, we have changed the original sentence from: “Convalescent plasma was not associated with a higher rate of SARS-CoV-2 RT-PCR clearance in nasopharyngeal swabs, suggesting that it does not provide a strong antiviral activity at this stage in patients with COVID-19.”

To:

“ The rate of SARS-CoV-2 RT-PCR clearance in nasopharyngeal swabs did not differ between study arms either, suggesting that the provision of convalescent plasma in this study did not provide enough antiviral activity in patients with COVID-19 at this stage.” (see Discussion section, 2^nd^ paragraph)

Regarding the Ct values (shown in Fig. 3B), as suggested in the first round of revisions, we evaluated the effect of conv. plasma on viral load (Ct) clearance. We consider that the comparison of the Ct ratios between day 3 (or day 7) and the baseline for each patient, provides a better representation of plasma effect size than comparing pooled mean or median Ct values; therefore we would rather keep Fig.3 as it is.

Lines 423-426: The lack of antibody positivity in transfused patients is curious. Does the group of patients who did not exhibit a rise in antibody level include patients who did not have antibody at enrollment? The higher neutralizing titers of patients with a longer symptom duration is consistent with other studies (see Garcia-Beltran et al. medRxiv).

R: We agree with the reviewer’s comment, the observation commented in that paragraph (and included in Results section, Immune response subgroup analysis) is correct. We expected that convalescent plasma would drive a significant increase in antibody concentrations in transfused patients, but 7 from 26 patients from the immediate plasma arm (27%) still did not have a positive SARS-CoV-2 IgG detection 72 hrs. after plasma infusion. All these patients did not have antibodies on admission either. This reinforces that we do not know which is the “dose” of convalescent plasma required to optimize antibody levels in recipients. We have re-ordered the sentences in the paragraph describing antibodies development for clarity (Results section, Immune response subgroup analysis, 2^nd^ paragraph).
